# Arginine Methyltransferase PRMT1 Regulates p53 Activity in Breast Cancer

**DOI:** 10.3390/life11080789

**Published:** 2021-08-05

**Authors:** Li-Ming Liu, Qiang Tang, Xin Hu, Jing-Jing Zhao, Yuan Zhang, Guo-Guang Ying, Fei Zhang

**Affiliations:** 1 Tianjin Medical University Cancer Institute and Hospital, Tianjin 300060, China; liuliming126@tmu.edu.cn (L.-M.L.); tangqiang678@tmu.edu.cn (Q.T.); huxin2018@tmu.edu.cn (X.H.); zhaojingjing678@tmu.edu.cn (J.-J.Z.); 2 National Clinical Research Center for Cancer, Tianjin 300060, China; 3 Tianjin’s Clinical Research Center for Cancer, Tianjin 300060, China; 4 Key Laboratory of Cancer Prevention and Therapy, Tianjin 300060, China; 5 Department of International Medical Services, Peking Union Medical College Hospital, Chinese Academy of Medical Sciences, Beijing 100005, China; yuanzhang@ibms.pumc.edu.cn

**Keywords:** breast cancer, PRMT1, p53, arginine methylation

## Abstract

The protein p53 is one of the most important tumor suppressors, responding to a variety of stress signals. Mutations in p53 occur in about half of human cancer cases, and dysregulation of the p53 function by epigenetic modifiers and modifications is prevalent in a large proportion of the remainder. PRMT1 is the main enzyme responsible for the generation of asymmetric-dimethylarginine, whose upregulation or aberrant splicing has been observed in many types of malignancies. Here, we demonstrate that p53 function is regulated by PRMT1 in breast cancer cells. PRMT1 knockdown activated the p53 signal pathway and induced cell growth-arrest and senescence. PRMT1 could directly bind to p53 and inhibit the transcriptional activity of p53 in an enzymatically dependent manner, resulting in a decrease in the expression levels of several key downstream targets of the p53 pathway. We were able to detect p53 asymmetric-dimethylarginine signals in breast cancer cells and breast cancer tissues from patients, and the signals could be significantly weakened by silencing of PRMT1 with shRNA, or inhibiting PRMT1 activity with a specific inhibitor. Furthermore, PRMT1 inhibitors significantly impeded cell growth and promoted cellular senescence in breast cancer cells and primary tumor cells. These results indicate an important role of PRMT1 in the regulation of p53 function in breast tumorigenesis.

## 1. Introduction

The protein p53 mainly functions as a transcriptional factor, regulating important cellular processes, such as cell cycle arrest, DNA repair, apoptosis, and senescence. It has been considered as one of the most frequently mutated genes in human cancers [1]. Responding to DNA damage or other types of stress signals, p53 rapidly accumulates and activates the transcription of an array of downstream targets to induce growth arrest, such as the cyclin-dependent kinase inhibitor *p21* and *GADD45A*, as well as the genes involved in apoptosis, including *BAX*, *BID* and *PUMA* [2].

It has been reported that the transcriptional activity of p53 can be modulated through diverse modifications, including phosphorylation, acetylation, sumoylation, and methylation, whose abnormity may contribute to tumorigenesis [3,4,5]. In normal conditions, p53 binds to E3 ubiquitin ligase MDM2, which leads to p53 degradation through the ubiquitin–proteasome pathway. However, when responding to stresses, the phosphorylation of S15/S20 at the N-terminal of p53 disrupts the interaction of p53 and MDM2, resulting in p53 stabilization and an elevation of p53 protein level in the nucleus. Many lysine and arginine residues in p53 can be modified by methylation. It has been reported that K370, K372, and K382 in the C-terminus of p53 are methylated by lysine methyltransferases (KMTs), including KMT3C (SMYD2), KMT7 (SET7/9), and KMT5A (SET8) [2,6]. While p53 arginine methylation has only been detected in one report, Jansson et al. showed that protein arginine methyltransferase 5 (PRMT5) methylated R333, R335, and R337 residues, and influenced the specificity of p53 target genes [7].

Arginine residues can be methylated by PRMTs, generating monomethyl-arginine (MMA), asymmetrical dimethyl-arginine (aDMA), or symmetrical dimethyl-arginine (sDMA). PRMTs have been proved as the crucial regulators involved in several fundamental cellular processes, including transcriptional regulation, DNA damage response, pre-mRNA splicing, cell signaling, and cell fate decision [8]. Lots of studies have confirmed that PRMTs are highly expressed in various types of cancers, and are associated with poor prognosis of cancer patients [9,10,11]. PRMT1 is a crucial member of the PRMTs family in mammalian cells, and is responsible for about 85% of the total cellular PRMT activity. It functions as a transcriptional co-activator via methylating histone H4R3 to form H4R3me2, which is generally associated with active transcription by recruiting other co-factors and facilitating the binding of transcription factors (TFs) [12]. PRMT1 also regulates transcription via direct methylation of TFs or cofactors, such as FOXO1 [13], RUNX1 [14], and C/EBPα [15].

Several cancer-associated arginine mutations have been found in p53, which alter the biochemical properties of p53. Arginine modification may provide additional mechanisms affecting p53 function. PRMT1 is responsible for the main activities of PRMTs and whether it affects and regulates p53 activity is worthy of further exploration. In the present study, we found that knockdown of PRMT1 activates the expression of multiple p53 targets and leads to cellular senescence and growth-arrest. PRMT1 can bind to p53 and further inhibit p53-mediated transcriptional activation. Furthermore, arginine-methylated p53 is also found in breast cancer cells and the methylation signal of p53 can be weakened by silencing of PRMT1 with shRNA or inhibiting PRMT1 activity with a specific inhibitor.

## 2. Materials and Methods

### 2.1. Cell Culture

MCF7, MDA-MB-231, HEK293T, H1299, and HCT116 p53^−/−^ cells were cultured in DMEM medium supplemented with 10% FBS and 100 U/mL penicillin and streptomycin. All cell lines were negative for mycoplasma contamination and shown to be negative.

Breast cancer tissue processing was performed as described previously [16]. The cells were resuspended in 50% Matrigel (Corning, Corning, NY, USA, 354234; diluted with the primary breast cancer medium) and 50 μL drops of Matrigel-cell suspension were seeded into 24-well plates [16]. After the Matrigel solidified at 37 °C for 1 h, 500 μL of primary medium was added. The medium was changed every 3 days and organoids were passaged every 1~2 weeks by incubating organoids for 10~15 min in TrypLE Express (Gibco, Dublin, Ireland, 12605010). The primary breast cancer medium was prepared as previously described with several modifications. Advanced DMEM/F12 (Gibco, Dublin, Ireland, 12634-010) was supplemented with 10 mM HEPES (Gibco, Dublin, Ireland, 15630080), 1× GlutaMAX (Gibco, Dublin, Ireland, 35050061), 1× B27 (Gibco, Dublin, Ireland, 17504-044), 50 ng/mL EGF (R&D, Minnesota, USA, 236-EG), 20 ng/mL FGF10 (Peprotech, London, UK, 100-26), 5 ng/mL FGF7 (Peprotech, London, UK, 100-19), 1.25 mM N-acetyl cysteine (Sigma, St. Louis, MO, USA, A9165), 5 mM nicotinamide (Sigma, St. Louis, MO, USA, N0636), 0.5 μM A83-01 (Tocris, Bristol, UK, 2939), 10 μM Y-27632 (MCE, New Jersey, USA, HY-10071), 500 ng/mL R-spondin 1 (Peprotech, London, UK, 120-38), 100 ng/mL Noggin (Peprotech, London, UK, 120-10C), 5 nM neuregulin 1 (Peprotech, London, UK, 100-03), 500 nM SB202190 (Sigma, St. Louis, MO, USA, S7067), 50 μg/mL primocin (Invivogen, San Diego, CA, USA, Ant-pm-1) and 1× penicillin/streptomycin.

Cells were treated with furamidine dihydrochloride (Fur; Sigma, St. Louis, MO, USA, SML1559), a specific inhibitor of PRMT1 [17]. The concentration and treating time are provided in the figure legends.

### 2.2. Antibodies

Antibodies for PRMT1 (07-404) and asymmetric dimethyl-arginine (ASYM25, anti-Rme2, 09-814) were obtained from Millipore; antibodies for p53 (ab1101) and p16 (ab108349) were purchased from Abcam; antibodies for p53 (sc-6243), p21 (sc-6246), GAPDH (sc-47724), and β-ACTIN (sc-47778) were from Santa Cruz; antibody for FLAG (PM020) was from MBL; antibody for FLAG (F3165) was from Sigma; antibodies for p21 (2947) and GADD45A (4632S) were from CST; and the antibody for HA (11867423001) was from Roche.

### 2.3. Plasmids

Plasmids used in this study, including PRMT1(WT)-pcDNA6, PRMT1(E153Q)-pcDNA6, PRMT1(WT)-pCMV-tag3B, p53-pcDNA6, and p53-Luc, were constructed as described previously [15,18]. PRMT1(WT) and PRMT1(E153Q) were cloned into pGEX4T-1 vectors. PRMT1(G80R)-pcDNA6 was generated using PCR-based site-directed mutagenesis. HA-tagged p53 was generated by inserting HA sequences downstream of the p53 coding region in the p53-pcDNA6 plasmid. Truncated mutants of p53 (ΔC 1-313 aa, ΔN 96-393 aa, and core 96-313 aa) were gifts from Prof. Ye Zhang (Institute of Basic Medical Sciences, Chinese Academy of Medical Sciences and Peking Union Medical College, Beijing, China). Primers used for constructing the PRMT1(G80R)-pcDNA6 plasmid were listed in Supplementary Table S1.

### 2.4. Establishment of PRMT1 Knockdown Cell Lines

PRMT1 knockdown cell lines were established as described previously [15]. Briefly, PRMT1 shRNAs were cloned into the pLKO.1 vector and control shRNA was a hairpin RNA designed against GFP. HEK293T cells were cotransfected with lentiviral constructs (psPAX2 and pMD2.G) and the indicated pLKO.1 plasmid. MCF7 and MDA-MB-231 cells were infected using lentivirus expressing shPRMT1 or shGFP. Infected cells were selected with puromycin. Then, Western blotting analysis was performed to determine the expression levels of PRMT1 in multiple monoclonal cultures. The primers are listed in Supplementary Table S1.

### 2.5. Establishment of PRMT1-Overexpressing Cell Lines

MCF7 cells were transfected with PRMT1(WT)-pcDNA6 or PRMT1(E153Q)-pcDNA6 plasmids using Lipofectamine 3000 (Invitrogen, L3000015). Transfected cells were selected with 10 μg/mL blasticidin at 48 h post-transfection. Then, Western blotting analysis was performed to determine the expression levels of overexpressed PRMT1 in multiple monoclonal cultures.

### 2.6. Western Blotting

Western blotting assay was performed as described previously [15]. Briefly, cells were lysed in RIPA buffer (50 mM HEPES (pH 7.4), 150 mM NaCl, 2 mM EDTA (pH 8.0), 2 mM EGTA (pH 8.0), 0.5% NP-40) containing protease inhibitor cocktail (Roche, 4693124001). Cell lysates were separated via SDS-PAGE and then transferred to PVDF membranes. The membranes were incubated with indicated primary antibodies at 4 °C overnight, followed by horseradish peroxidase (HRP)-labelled secondary antibodies at room temperature for 2 h. Protein band intensities were measured using the ImageJ software (ver. 1.53a, NIH, Bethesda, MD, USA). Original Western blotting images are shown in Supplementary File S1.

### 2.7. GST-Pull Down

GST-tagged PRMT1-WT/E153Q-pGEX4T-1 vectors were expressed in *E.coli* BL21. GST-PRMT1-WT/E153Q proteins were purified using Glutathione Sepharose 4B (GE, 17-0756-01). Washed PRMT1-WT/E153Q-beads were incubated with cell lysates from HEK293T transfected with p53-pcDNA6 vector at 4 °C overnight. Then, the beads were washed 4–6 times with RIPA buffer, resuspended in 30 μL 2× SDS loading buffer, and detected by Western blotting.

### 2.8. Co-Immunoprecipitation

Co-immunoprecipitation (Co-IP) assay was performed as described previously [19]. Cells were lysed using RIPA buffer for 1 h at 4 °C, followed by centrifugation at 12,000× *g* rpm for 20 min. Supernatant was rotated at 4 °C overnight with anti-FLAG magnetic beads (Bimake, B26101) or specific antibodies and protein A/G agarose beads. The pellets were washed 3–5 times using RIPA buffer, followed by the addition of 30 μL 2× SDS loading buffer and boiled.

### 2.9. qRT-PCR

A qRT-PCR assay was performed as described previously [19] and primers are shown in Supplementary Table S1.

### 2.10. Reporter Gene Assay

Reporter gene assay was performed as previously described [20]. Briefly, cells were seeded in 24-well plates and co-transfected with expression plasmids, firefly luciferase plasmid, and Renilla luciferase plasmid. Firefly and Renilla luciferase activities were measured using the Dual Luciferase Reporter Assay System (Promega) 48 h after transfection and normalized to Renilla activity.

### 2.11. Colony Formation Assay

Cells were seeded in 6-well plates at a density of 800 cells/well. After incubation for about 12 days, cells were washed with PBS twice and then fixed and stained with 0.1% crystal violet.

### 2.12. CCK-8 Assay

Cells were seeded at a density of 2 × 10^3^ cells/well in triplicate in 96-well plates. Proliferation was assessed using CCK-8 (APExBIO, K1018). Briefly, diluted CCK-8 (1:10) solution was added to each well and incubated at 37 °C for 4 h at various time points. Subsequently, cell proliferation was assessed by measuring the absorbance at 450 nm. Wells without cells (only medium) were used as the blanks.

### 2.13. Senescence-Associated β-Galactosidase Staining

Senescence-associated β-galactosidase (SA-β-gal) catalyzes the hydrolysis of X-gal, which produces a blue color in senescent cells. SA-β-gal staining was carried out as reported previously [21]. Cells were washed with PBS twice and then fixed for 10 min at room temperature with 4% paraformaldehyde. Subsequently, cells were washed with PBS twice and then incubated with the staining solution (1 mg/mL X-gal, 40 mmol/L citric acid/sodium phosphate buffer (pH 6.0), 5 mM potassium ferrocyanide, 5 mM potassium ferricyanide, 150 mM sodium chloride, and 2 mM magnesium chloride) overnight at 37 °C.

### 2.14. Annexin V-FITC/Propidium Iodide Staining

Annexin V-FITC/Propidium Iodide (PI) Apoptosis Detection Kit (BD, 556547) was used to analyze the percentage of apoptotic cells. The experiment was conducted as the manufacturer’s instruction. Briefly, 5 × 10^5^ cells were harvested and washed twice with PBS. Cells were resuspended in 100 μL 1× binding buffer and incubated with 5 μL of FITC-conjugated annexin V and 5 μL of PI for 10 min at room temperature in the dark, then 400 μL 1 × binding buffer was added. Samples were analyzed immediately. The results were obtained with a flow cytometer (BD, FACSCanto II) and analyzed with FlowJo software.

### 2.15. Patient Samples

This study was approved by the Institutional Ethics Committee of Tianjin Medical University Cancer Institute and Hospital (Tianjin, China). The written informed consent was obtained from the participants. Primary breast cancer tissues were obtained from patients treated at the Tianjin Medical University Cancer Institute and Hospital, China.

### 2.16. RNA-seq

RNA-seq data was obtained from the NCBI Gene Expression Omnibus database (http://www.ncbi.nlm.nih.gov/geo/, accessed on 11 July 2021, accession number: GSE121168). The method for the data analysis was described as our previous study [15].

### 2.17. PRMT1 Expression Analysis

Fragments per kilobase million (FRKM) normalized expression profile data of breast cancer samples (including 113 normal and 998 tumor tissues) were downloaded from the Cancer Genome Atlas (TCGA) database (https://www.cancer.gov/about-nci/organization/ccg/research/structural-genomics/tcga, accessed on 11 July 2021), and the data of PRMT1 gene were extracted. Then, FPKM values were transformed into transcripts per kilobase million (TPM) values. Boxplots and scatter plots were generated to calculate the expression of PRMT1 in breast cancer and normal breast tissues. The Wilcoxon rank-sum test was adopted.

### 2.18. Disease Specific Survival Analysis

The expression profile data of breast cancer patients (n = 998) with complete clinical information were downloaded from TCGA database. The patients were divided into PRMT1 high- and low-expression groups according to the optimal cutoff value calculated using the “survminer” package (https://CRAN.R-project.org/package=survminer, accessed on 11 July 2021) [22,23]. The Kaplan–Meier method was used to assess the efficiency of disease-specific survival (DSS) in high- and low-expression patients. The log-rank test was used to assess statistical significance at *p* < 0.05.

### 2.19. Statistical Analysis

All experiments were carried out in independent triplicate. Results of experiments were shown as mean ± SD. An unpaired Student’s *t*-test was used to analyze differences between two groups. One-way analysis of variance (ANOVA) was used to analyze multiple comparisons using GraphPad Prism software (version 7.0; GraphPad Software Inc., San Diego, CA, USA). Tukey’s test was used for the pair group’s comparison. A *p*-value less than 0.05 was considered statistically significant.

## 3. Results

### 3.1. PRMT1 Knockdown Leads to Growth Arrest and Senescence

**Figure 1:**
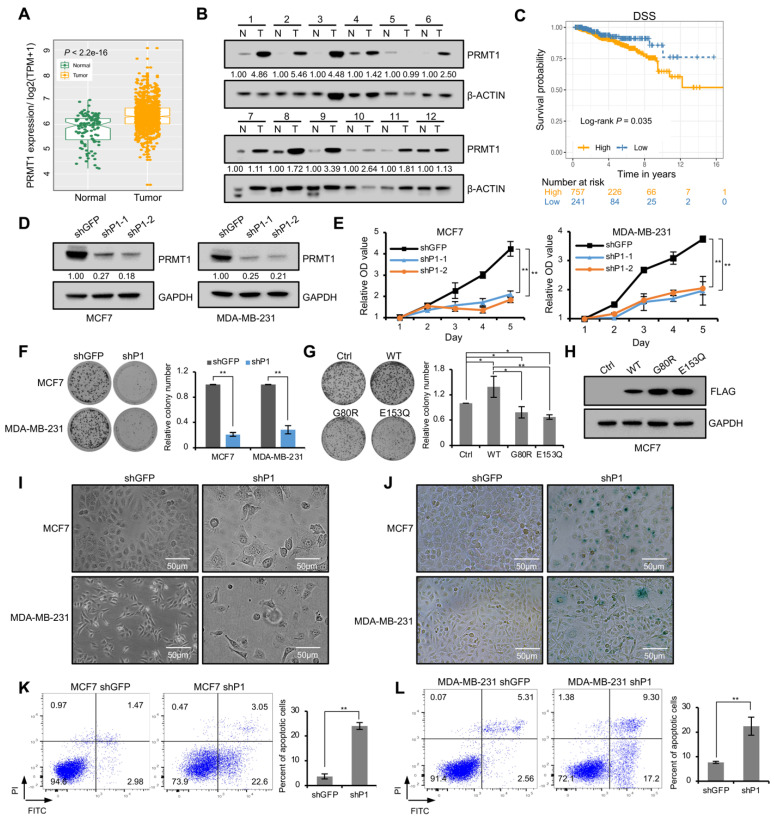
Knockdown of PRMT1 promoted growth-arrest and cellular senescence in breast cancer cells. (**A**) The box plots were analyzed from the TCGA database to compare the expression of PRMT1 in cancers and normal breast samples; *p* < 0.05. (**B**) Western blotting analysis showing PRMT1 levels in 12 pairs of breast tumor (T) and paired normal tissues (N). β-ACTIN served as a reference gene. PRMT1 expression was normalized by the reference gene and the relative expression of PRMT1 was shown as the ratio of tumor tissues relative to their paired normal tissue. (**C**) The prognostic value of mRNA level of PRMT1 in breast cancer patients. DSS: disease-specific survival. *p* < 0.05. (**D**) The expression of PRMT1 was measured using Western blotting in shPRMT1 (shP1-1 and shP1-2) or shGFP MCF7 and MDA-MB-231 cells. GAPDH served as a reference gene. The expression of PRMT1 relative to shGFP was presented. (**E**) CCK-8 assays were performed to value the proliferation ability of MCF7 and MDA-MB-231 cells with PRMT1 knockdown. Data represent the mean ± SD from three independent experiments. (**F,G**) Colony formation assays shown in shPRMT1 (shP1) or shGFP MCF7 and MDA-MB-231 cells (**F**), or in MCF7 cells transfected with expression plasmids of vector (Ctrl), wild type PRMT1 (WT), or mutated PRMT1 (G80R and E153Q) (**G**). Quantification shown on the right. Data represent the mean ± SD of triplicate cultures. (**H**) The expression levels of PRMT1 plasmids were measured by Western blotting using anti-FLAG antibody in MCF7 cells. (**I**) Cell morphology of shPRMT1 or shGFP MCF7 and MDA-MB-231 cells, scale bar: 50 μm. (**J**) shPRMT1 and shGFP MCF7 and MDA-MB-231 cells were stained by X-gal, scale bar: 50 μm. (**K,L**) The analysis of intensity of annexin V-FITC/PI staining in shGFP and shPRMT1 MCF7 (**K**) and MDA-MB-231 cells (**L**). Annexin V (+)/PI (+/−) populations (%) were regarded as apoptosis. Quantification shown on the right. The results were presented as mean ± SD from three independent experiments. *, *p* < 0.05; **, *p* < 0.01.

The analysis of cancer vs. normal breast samples in TCGA database revealed that PRMT1 expression level was significantly elevated in breast cancer patients compared with the adjacent normal tissues (Figure 1A). PRMT1 expression was verified using 12 paired breast tumor and normal tissues by Western blotting, and the result revealed that the expression levels of PRMT1 were significantly increased in 9 out of 12 breast tumor samples (T) compared with paired breast normal samples (N) (Figure 1B), which was consistent with our recent report [20]. The relationship between PRMT1 expression and the clinicopathologic characteristics of breast cancer was further explored. Univariate Cox regression analysis demonstrated that patients with high levels of PRMT1 had aggressive clinicopathological features, including advanced T stage and high TNM stage (Table 1). Besides, multivariate analysis indicated that the high PRMT1 expression in breast cancer tissues could be an independently prognostic marker of poor prognosis (Table 1). We also explored the predictive efficiency of PRMT1 in the survival of patients with breast cancer. The log rank test analyses revealed that the increased PRMT1 mRNA was significantly negatively associated with DSS of the patients with breast cancer (Figure 1C).

To further determine the function of PRMT1, we established PRMT1 knockdown cell lines via lentiviral shRNA infection in breast cancer cells, MCF7 and MDA-MB-231. The decreased expression of PRMT1 was confirmed using Western blotting analysis (Figure 1D). CCK-8 assays suggested that PRMT1 knockdown attenuated the proliferation ability of MCF7 and MDA-MB-231 cells (Figure 1E), which was further verified by colony formation assays (Figure 1F). To determine whether the methyltransferase activity is required for PRMT1-mediated regulation of cell growth, PRMT1 WT or two mutants (G80R and E153Q) with impaired enzymatical activity were overexpressed in MCF7 cell lines [24,25,26,27]. The results showed that the wild-type PRMT1, but not the inactive mutants, promoted cell proliferation (Figure 1G,H), which suggested that the methyltransferase activity was required for PRMT1 to promote cell proliferation. In addition, shPRMT1 induced obvious changes in cell morphology; the cells were flattened and enlarged (Figure 1I). The morphological changes are often associated with cellular senescence [28,29,30]. Moreover, these flat cells were stained positively for SA-β-gal (Figure 1J), which is widely used as a senescence marker, demonstrating that PRMT1 was involved in cell senescence. In addition, annexin V-FITC/PI staining showed that the proportions of early and late apoptotic cells were significantly increased in shPRMT1 MCF7 and MDA-MB-231 cells compared with the shGFP control (Figure 1K,L). These results revealed that PRMT1 inhibits cellular senescence and accelerates proliferation of breast cancer cells.

### 3.2. PRMT1 Participates in the Regulation of p53 Target Genes

**Figure 2:**
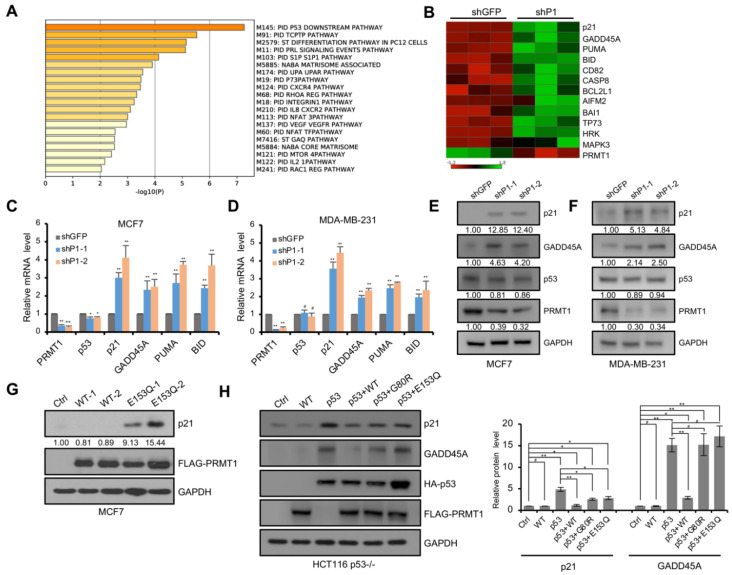
PRMT1 regulated the expression of p53 targets in breast cancer cells. (**A**) The analysis of upregulated genes by Metascape showing the top 20 canonical pathways in shPRMT1 MDA-MB-231 cells compared with the shGFP control; *p* < 0.05. (**B**) Heatmap of p53 pathway genes in the upregulated genes (*p* < 0.05) analyzed with KOBAS and visualized using Matrix2png in shPRMT1 MDA-MB-231 cells. Green and red depict high and low expression levels, respectively, as indicated by the scale bar. (**C**–**F**) The expression levels of PRMT1, p53, and p53 targets were measured by RT-PCR (**C**,**D**) and Western blotting (**E**,**F**) in shPRMT1 (shP1-1 and shP1-2), and shGFP MCF7, and MDA-MB-231 cells. (**G**) Detection of indicated genes expression by Western blotting in MCF7 cells transfected with plasmids expressing wild-type (WT-1 and WT-2, two monoclonal cell lines) or mutated PRMT1 (E153Q-1 and E153Q-2, two monoclonal cell lines). (**H**) Western blotting analysis of the expression of indicated proteins in HCT116 p53^−/−^ cells transfected with PRMT1 (WT), p53, p53 and PRMT1-WT (p53 + WT), or p53 and PRMT1-mutants (p53 + G80R and p53 + E153Q). Quantification shown on the right. The results were presented as mean ± SD from three independent experiments. #, *p* > 0.05; *, *p* < 0.05; **, *p* < 0.01.

**Figure 3:**
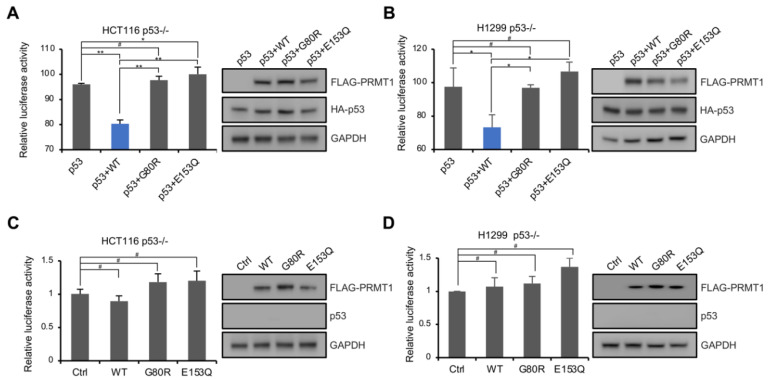
PRMT1 repressed the transcriptional function of p53. (**A**–**D**) Reporter gene assays were performed in HCT116 p53^−/−^ (**A**,**C**) and H1299 p53^−/−^ cells (**B**,**D**) transfected with p53-Luc reporter plasmid, together with plasmids expressing p53 and PRMT1-WT/mutant expression plasmids (**A**,**B**), or reporter plasmid and PRMT1-WT/mutant plasmids (**C**,**D**). The expression of FLAG-tagged PRMT1, HA-tagged p53, or endogenous p53 is shown on the right. Data were presented as the means ± SD and analyzed using one-way ANOVA. #, *p* > 0.05; *, *p* < 0.05; **, *p* < 0.01.

To identify the mechanism by which PRMT1 affects breast cancer cell proliferation and senescence, we analyzed the published RNA-seq dataset in PRMT1-knockdown MDA-MB-231 cells [15]. The pathway enrichment analysis using Metascape (https://metascape.org, accessed on 11 July 2021) [31] revealed that the upregulated genes in PRMT1-knockdown cells were significantly enriched in the p53 downstream pathway (Figure 2A). KOBAS analysis (http://kobas.cbi.pku.edu.cn/kobas3, accessed on 11 July 2021) [32], visualized using Matrix2png (https://matrix2png.msl.ubc.ca, accessed on 11 July 2021) [33], showed the enriched p53 pathway genes, including *p21*, *GADD45A*, *PUMA*, *BID*, *CD82*, *CASP8*, *BCL2L1*, *AIFM2*, *BAI1,* and *TP73* (Figure 2B). Besides, apoptosis pathway genes, *HRK* and *MAPK3*, were also activated. We next investigated the potential role of PRMT1 in the regulation of the p53 pathway. qRT-PCR analysis showed that *p21* and *GADD45A*, involved in cell cycle arrest, were induced in shPRMT1 MDA-MB-231 and MCF7 cell lines (Figure 2C,D), and *PUMA* and *BID*, implicated in p53-induced apoptosis, were also activated (Figure 2C,D). Similarly, Western blotting assays showed that the protein levels of p21 and GADD45A were remarkably increased in shPRMT1 cells compared with shGFP cells (Figure 2E,F). However, the mRNA and protein levels of p53 exhibited no obvious changes in MDA-MB-231 cells, but slightly decreased in MCF7 cells (Figure 2C–F). We also constructed MCF7 cells with stable expression of PRMT1-WT and PRMT1-E153Q genes. The exogenous expression of PRMT1-E153Q mutants (lanes 4 and 5 in Figure 2G), but not the PRMT1-WT (lanes 2 and 3 in Figure 2G), in MCF7 cells activated p21 expression. The inactive PRMT1-E153Q mutant may compete with the wild-type for binding to target factors and thus inhibit the function of the wild-type. These data indicated that the downregulation of the p53 pathway by PRMT1 depends on its enzymatic activity. Similarly, the co-overexpression of exogenous PRMT1-WT and p53 exhibited stronger p53 target inhibition than those of the co-overexpression of PRMT1 mutants (G80R and E153Q) and p53 in p53-deficient HCT116 cells (Figure 2H). In dual-luciferase reporter assays, p53-null HCT116 and H1299 cells were transfected with p53-Luc reporter plasmid which contains multiple copies of the p53-binding consensus sequence, together with plasmids expressing p53 and/or PRMT1-WT/mutant, and the luciferase activities were measured. The data showed that the co-expression of wild-type PRMT1, but not mutated PRMT1, impaired p53-mediated transcriptional activation (Figure 3A,B). In addition, PRMT1 lost the inhibitory effect on p53-Luc reporter gene in cells that didn’t co-express p53, demonstrating that p53 was the direct target of PRMT1 for transcriptional inhibition (Figure 3C,D). These results indicated that PRMT1 inhibits p53 transcriptional activity in an enzymatically dependent manner.

### 3.3. p53 Arginine Methylation in Cells

**Figure 4:**
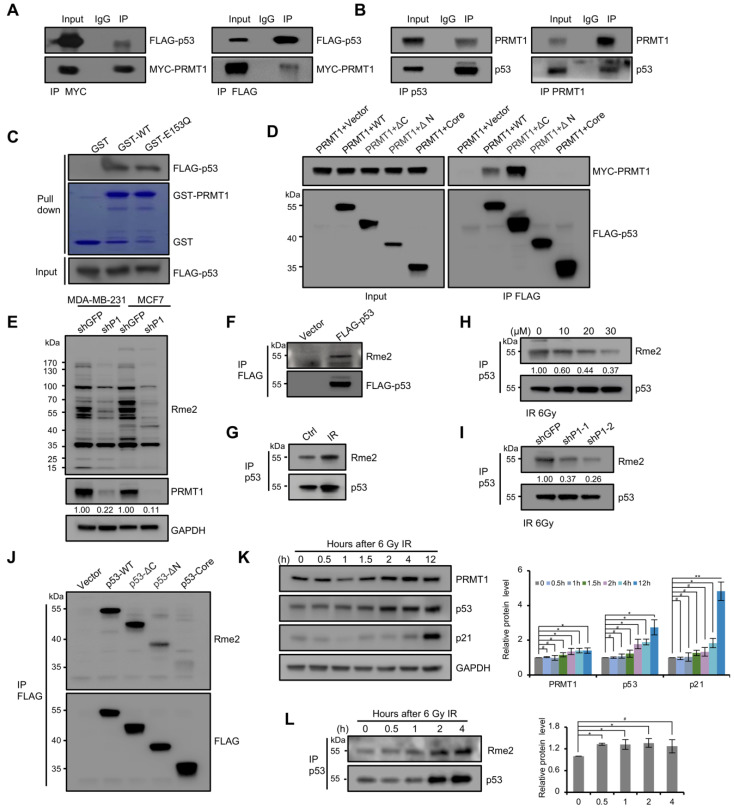
p53 was methylated on arginine residues in cells. (**A**) Co-IP assays were performed in HEK293T cells transfected with MYC-PRMT1 and FLAG-p53 plasmids using anti-MYC (**left**) and anti-FLAG (**right**) antibodies, and then immunoblotting using anti-FLAG and anti-MYC antibodies. (**B**) Co-IP assays were performed in MCF7 cells using anti-p53 (**left**) and anti-PRMT1 (**right**) antibodies. (**C**) GST-pulldown assay was performed using FLAG-p53 vector transfected HEK293T cell lysates incubated with GST, GST-fused PRMT1-WT, or -E153Q, followed by Western blotting with anti-FLAG antibody. The protein levels of GST and GST-fused PRMT1 were shown by Coomassie Brilliant Blue staining. (**D**) Domain mapping of p53 region interaction with PRMT1. FLAG-p53 or its truncations were co-expressed with MYC-PRMT1 in HEK293T cells and precipitated using anti-FLAG beads. Co-IPed PRMT1 was detected by Western blotting using anti-MYC antibody. Left, input. (**E**) Whole cell-lysates from shPRMT1 and shGFP MDA-MB-231/MCF7 cells were immunoblotted with anti-Rme2 antibody. (**F**–**I**) Detection of p53 arginine methylation by Western blotting using anti-Rme2 antibody. (**F**) FLAG-p53 was immunoprecipitated with anti-FLAG antibody from HEK293T cells expressing FLAG-p53. (**G**) p53 was immunoprecipitated with anti-p53 antibody from MCF7 cells after 2 h of IR treatment (6 Gy). (**H**) p53 was precipitated using anti-p53 antibody from MCF7 cells after 2 h of 6 Gy IR; cells were treated with increasing concentrations of Fur (0, 10, 20 or 30 μM) for 24 h. Fur: Furamidine dihydrochloride. (**I**) p53 was precipitated using anti-p53 antibody from shPRMT1 or shGFP MCF7 cells after 2 h of 6 Gy IR. (**J**) Domain mapping of the p53 methylation region. FLAG-tagged p53 truncations were expressed in HEK293T cells and precipitated using anti-FLAG magnetic beads. Methylation was analyzed by Western blotting using anti-Rme2 antibody. (**K**) Detecting the protein levels of PRMT1, p53, and p21 by Western blotting in MCF7 cells after exposure to 6 Gy IR. Quantification shown on the right. Data were presented as mean ± SD from three independent experiments. (**L**) Western blotting using anti-Rme2 antibody for the detection of the methylated p53 purified from MCF7 cells after 6 Gy IR. The arginine methylation levels were normalized by p53.Quantification shown on the right. Data were presented as mean ± SD from three independent experiments. #, *p* > 0.05; *, *p* < 0.05; **, *p* < 0.01.

To investigate whether PRMT1 can directly regulate p53 function, we next examined the potential physical relationship between PRMT1 and p53. Co-IP assays were performed using HEK293T cell lysate co-transfected with MYC-tagged PRMT1 and FLAG-tagged p53 with MYC/FLAG antibodies. The results showed that FLAG-p53 and MYC-PRMT1 can immunoprecipitate each other (Figure 4A), which was consistent with earlier literature [34]. The endogenous interaction between PRMT1 and p53 was also confirmed by detecting p53 and PRMT1 precipitates using the specific antibodies in MCF7 cells (Figure 4B). Furthermore, p53 from transfected HEK293T lysates was pulled down by bacterially purified GST-PRMT1-WT and GST-PRMT1-E153Q, but not by GST alone (Figure 4C). These results indicated that PRMT1 directly interacts with p53. To determine which region of p53 bound to PRMT1, we precipitated a series of p53 deletion mutants from FLAG-tagged p53 and MYC-tagged PRMT1 co-overexpressed HEK293T cells. Western blotting showed that the full-length p53 and ΔC (1-313 aa) obviously interacted with PRMT1, but the ΔN (96-393 aa) and core (96-313 aa) deletions lost the ability to bind to PRMT1 (Figure 4D). This finding was consistent with the previous report [34], demonstrating that the N-terminal region (1-95 aa) was required for p53–PRMT1 interaction.

PRMT1 is the main PRMT catalyzing the aDMA of target proteins. We found that the overall levels of aDMA signals were decreased in shPRMT1 MCF7 and MDA-MB-231 cells by using the aDMA-specific antibody (anti-Rme2) (Figure 4E). We next tested whether p53 was methylated at arginine residues in cultured cells. The precipitate of FLAG-p53 from transfected HEK293T cells was detected using anti-Rme2 antibody, and an obvious signal was observed at the size of the p53 protein (Figure 4F). Besides, endogenous p53 purified from MCF7 cells treated with 6 Gy ionizing radiation (IR) was also methylated (Figure 4G), and the methylation signal was seriously reduced in the presence of the PRMT1-specific inhibitor furamidine dihydrochloride (Fur) (Figure 4H); shPRMT1 also decreased the methylation level of p53 in MCF7 cells (Figure 4I). These data indicated that PRMT1 modulates the arginine methylation of p53 in cells. We next identified the methylation region of p53, through immunoprecipitation of truncated mutants of p53 from transfected HEK293T cells. Western blotting with anti-Rme2 antibody showed that full-length p53 and ΔC (1-313 aa) were strongly methylated in IPed p53, and relatively weak signals were detected in the ΔN (96-393 aa) and core (96-313 aa) mutants (Figure 4J). The low intensity of methylation signals in the ΔN and core deletions may be due to the loss of the ability to bind to PRMT1, suggesting that arginine methylation of p53 occurs at least in the core fragment (96-313 aa) containing 19 arginine residues out of the total 26.

We next tested whether PRMT1 modulated p53 function in response to DNA damage; the levels of indicated proteins were analyzed by Western blotting in MCF7 cells following 6 Gy IR. As shown in Figure 4K, the protein level of p53 was enhanced post-radiation. With the accumulation of p53 protein, p21 expression was activated later. Unexpectedly, PRMT1 was also elevated in response to radiation. Besides, an elevated arginine methylation signal of p53 was observed in MCF7 cells treated with radiation (Figure 4L). These data suggested that PRMT1-mediated methylation may be involved in regulation of p53 function in response to DNA damage. Collectively, these results indicated that the function of p53 is regulated by arginine methylation under physiological condition, and PRMT1 may be involved in this process.

### 3.4. p53 Arginine Methylation in Primary Breast Cancer Tissues

**Figure 5:**
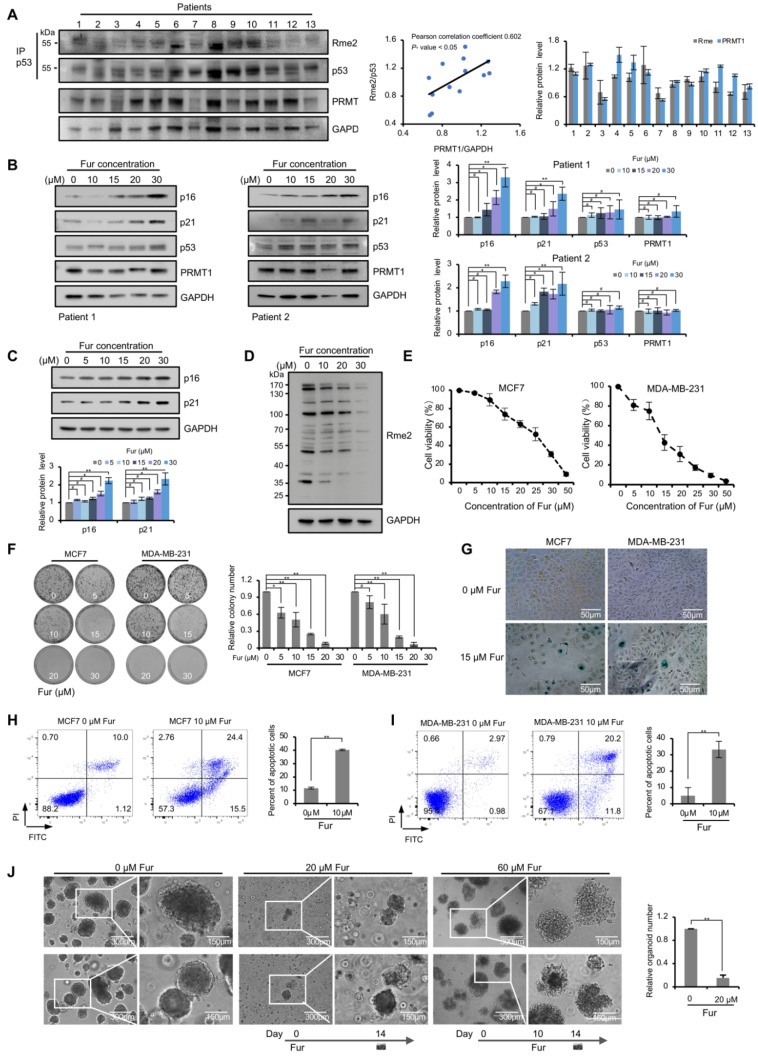
p53 response and cellular phenotypes in PRMT1 inhibitor-treated breast cancer cells. (**A**) Western blotting assays showing the arginine methylation levels of p53 precipitated with anti-p53 antibody from 13 breast cancer tissues (**top**). PRMT1 and GAPDH levels in 13 breast cancer tissues (**bottom**). PRMT1 expression levels were normalized by GAPDH. The arginine methylation levels were normalized by p53. The correlation analysis of Rme2 and PRMT1 protein levels presented in the middle (*p* < 0.05). Quantification shown on the right. Data were presented as mean ± SD. (**B**) The levels of indicated proteins were analyzed by Western blotting in primary breast cancer cells treated for 72 h with Fur (0, 10, 15, 20 and 30 μM). Quantification shown on the right. Data were presented as mean ± SD from three independent experiments. (**C**) The levels of indicated proteins were analyzed by Western blotting in MCF7 cells treated for 72 h with Fur (0, 5, 10, 15, 20 and 30 μM). Quantification shown on the bottom. Data were presented as mean ± SD from three independent experiments. (**D**) Whole cell lysates from MCF7 cells treated for 24 h with Fur were immunoblotted with anti-Rme2 antibody. (**E**) The viability of MCF7 and MDA-MB-231 cells treated with Fur for 72 h was detected through CCK-8 assays. Data were presented as mean ± SD from n = 3 independent experiments. (**F**) Colony formation assays shown in MCF7 and MDA-MB-231 cells treated with Fur for 12 days. Quantification shown on the right. Data were presented as mean ± SD from three independent experiments. (**G**) SA-β-gal staining of MCF7 and MDA-MB-231 cells treated with Fur for 48 h. (**H,I**) Flow cytometry analysis of the intensity of annexin V-FITC/PI staining in MCF7 (**H**) and MDA-MB-231 cells (**I**) treated with Fur (10 μM) for 48 h. Annexin V (+)/PI (+/−) populations (%) were regarded as apoptosis. Quantification shown on the right. Data were presented as mean ± SD from three independent experiments. (**J**) Organoid formation ability of primary breast cancer cells treated with Fur. In the middle panel, cells were treated with 20 μM Fur at day 0. In the right panel, cells were treated with 60 μM Fur at day 10. Quantification shown on the right. Data were presented as mean ± SD from n = 3 independent experiments. Fur: Furamidine dihydrochloride. #, *p* > 0.05; *, *p* < 0.05; **, *p* < 0.01.

Next, we assessed whether p53 arginine methylation occurs in breast cancer patients. The methylation levels of p53, precipitated from the samples of breast cancer patients, were investigated using anti-Rme2 antibody. As shown in Figure 5A, p53 arginine methylation signals were observed in almost all breast cancer tissues, demonstrating that p53 arginine methylation did indeed occur in breast cancer patients, which may be critically involved in the development and progression of breast carcinoma.

The protein levels of PRMT1 were also analyzed using the above samples, and we found that the strength of p53 methylation signals were partly positively correlated with the PRMT1 protein levels (5 out of 13, sample 3, 7, 8, 9 and 13) in patients (Figure 5A). The role of PRMT1 in regulating p53 function was further investigated in primary breast cancer cells. Two samples of primary cells were treated with different concentrations of Fur. The cells displayed obvious senescence phenotypes after undergoing treatment for three days. The protein level of p16, a biomarker for cellular senescence, was highly induced in these primary cells, and p21 level also showed an obvious increase (Figure 5B). Besides, p16 and p21 were also obviously induced in Fur-treated MCF7 cells (Figure 5C).

### 3.5. PRMT1 Inhibitor Blocks Growth and Induces Death of Breast Cancer Cells

We next tested the anti-cancer potency of PRMT1 inhibitor Fur in breast cancer cell lines and primary cancer cells. Firstly, the effect of Fur on PRMT1 enzymatic activity was validated. As shown in Figure 5D, Fur obviously blocked the overall levels of aDMA which was mainly catalyzed by PRMT1, suggesting the inhibitory effect on PRMT1 activity. CCK-8 and colony formation assays were conducted in MCF7 and MDA-MB-231 cells after Fur treatment. As shown in Figure 5E,F, Fur markedly decreased the proliferation and survival of breast cell lines. We further evaluated the effect of Fur on cellular senescence. SA-β-gal staining showed more senescent cells in the Fur-treated group than the control group (Figure 5G). In addition, annexin V-FITC/PI staining also revealed an increase in the apoptotic cell population in MCF7 and MDA-MB-231 cells following incubation with Fur (Figure 5H,I). Besides, we also evaluated the sensitivity of primary breast cancer cells to Fur drugs. The effect of Fur on cell growth was detected before and after the formation of primary breast cancer organoids, which was similar to preventive and rescue treatment. As depicted in Figure 5J, in the preventive group, few spheroids were formed in Fur-treated group. In the rescue group, Fur was added after drug-free growth of 10 days. Treated spheroids showed loosely-attached structure, and organoids dispersed into individual cells and then broke into pieces after several days of Fur-treatment. Taken together, these data revealed that PRMT1 inhibitor blocks cancer cell growth and induces cellular senescence and death.

## 4. Discussion

Recently, we have reported that PRMT1 is aberrantly expressed in breast cancer patients. PRMT1 promotes cell proliferation by activating the expression of *CCND1*, which partially explains the cell growth arrest induced by silencing of PRMT1 [15]. However, the mechanism of shPRMT1-mediated cellular apoptosis and senescence remains incompletely understood. Previous studies have shown that low PRMT1 expression in non-MYCN-amplified neuroblastoma cells promotes p53-mediated cellular senescence and migration activity [35]. Besides, PRMT1, as a co-activator of p53, can function independently or cooperatively with p300 and CARM1 (another PRMTs member) in p53-induced activation of *GADD45A* [34]. When detecting p53 expression in PRMT1 knockdown cells, we found that the expression level of p53 was slightly downregulated in MCF7 cells, which is consistent with the observation in MDA-MB-231 cells in our previous study [15]. However, the expression of p53 in MDA-MB-231 cells did not change after PRMT1 was silenced in this study, and the difference may result from the very weak effect of PRMT1 on p53 expression. In this research, we found that PRMT1 can impair the function of p53 in transcriptional activation, leading to the downregulation of several key genes in the p53 pathway. Furthermore, p53 is methylated at arginine residues in breast cancer cells, and the methylation signal of p53 can be attenuated by PRMT1 silencing or inhibiting its activity with the specific inhibitor. These results show an important role of PRMT1 in the post-translational regulation of p53.

Inactivation of the p53 tumor suppressor is frequently observed in human cancers. Among multiple mechanisms regulating p53, posttranslational modification (PTM) represents an efficient and widespread type, affecting the stability, localization, and binding partners of p53. Methylation at lysine and arginine residues is a simple but important epigenetic mark with complicated effects on p53 function. p53 K372 is methylated by SET7/9, resulting in the enhanced stability of p53 protein, but SET8-mediated K382 methylation and SMYD2-mediated K370 methylation lead to the attenuation of p53-regulated transcriptional activation [2,6]. To date, only one member of PRMT family, PRMT5, has been reported to methylate p53 at R333, R335, and R337 residues in the tetramerization domain, affecting the target gene specificity of p53 [7]. Besides, methylation of R110, R209, and R213 in p53 has also been confirmed by mass spectrometry analysis [36]. We found that multiple regions of p53 are modified by aDMA, and the methylation signal may be catalyzed by PRMT1, since the signal is seriously impaired by the PRMT1 inhibitor or PRMT1 silencing. However, further efforts are warranted to understand the detailed molecular mechanism of the effect of methylation on p53 function.

By analyzing p53 genetic alterations using cBioPortal (http://cbioportal.org, accessed on 11 July 2021) [37,38,39], we observed a high mutation rate in arginine residues of p53 protein in breast carcinoma patients, including R248, R273, R175, R342, R280, R282, R196, R337, R249, and R306 (ranked according to the mutation frequency) (Supplementary Figure S1). Most of the arginine mutations lie in the DNA-binding domain, resulting in the attenuation of the DNA binding ability of p53. We speculate that the methylation of arginine residues in p53 protein may have a similar effect on their mutation. Therefore, those mutated arginine residues in p53 protein present the potential for regulation by epigenetic modification. Unlike genetic mutation, epigenetic regulation is more flexible. Importantly, the methylation of R337, R335, and R213 at p53 has already been reported [7,36]. In this report, we found that PRMT1-mediated p53 methylation inhibits its transcriptional activity, and the identification of arginine methylation sites will be an important issue in our future investigation. In addition, we also revealed that the PRMT1 specific inhibitor Fur blocks cell growth and induces apoptosis and senescence in breast cancer cell lines and primary cells, demonstrating the antitumor effects of Fur on breast cancer cells. However, the efficacy of Fur in breast cancer patients needs to be further explored.

## Figures and Tables

**Table 1:** Univariate and multivariate Cox regression analysis of clinicopathological parameters and independent associated with prognosis, based on the TCGA database.

	Univariate Cox Regression Analysis		Multivariate Cox Regression Analysis	
	*p*-Value	Hazard Radio	*p*-Value	Hazard Radio
Age (<=65 vs >65)	0.402	1.247 (0.745–2.087)	0.135	1.487 (0.883–2.502)
Gender (female vs male)	0.764	1.353 (0.187–9.772)	0.870	0.846 (0.116–6.202)
Stage (I + II vs III + IV)	<0.001	3.826 (2.424–6.040)	0.048	1.811 (1.004–3.264)
T (T1 vs T2–4)	0.024	1.997 (1.096–3.640)	0.736	1.117 (0.585–2.134)
N (N0 vs N1–3)	<0.001	3.415 (1.986–5.870)	0.012	2.284 (1.197–4.361)
M (M0 vs M1)	<0.001	9.868 (5.293–18.399)	<0.001	4.227(2.100–8.512)
PRMT1 (Low vs High)	0.017	2.255 (1.158–4.392)	0.036	2.060 (1.048–4.052)

*p* < 0.05 was considered statistically significant.

## Data Availability

The data presented in this study are available on request from the corresponding author.

## Supplementary Materials

The following are available online at https://www.mdpi.com/article/10.3390/life11080789/s1; Supplementary Table S1: Primers used in this paper; Supplementary File S1: Original Western blotting images shown in the manuscript; and Supplementary Figure S1: Mutation analysis of p53 gene in breast cancer patients.

## References

[1] Bykov V.J.N., Eriksson S.E., Bianchi J., Wiman K.G. (2018). Targeting mutant p53 for efficient cancer therapy. Nat. Rev. Cancer.

[2] Aubrey B.J., Kelly G.L., Janic A., Herold M.J., Strasser A. (2018). How does p53 induce apoptosis and how does this relate to p53-mediated tumour suppression?. Cell Death Differ..

[3] Dai C., Gu W. (2010). p53 post-translational modification: Deregulated in tumorigenesis. Trends Mol. Med..

[4] Meek D.W., Anderson C.W. (2009). Posttranslational Modification of p53: Cooperative Integrators of Function. Cold Spring Harb. Perspect. Biol..

[5] Brooks C.L., Gu W. (2011). The impact of acetylation and deacetylation on the p53 pathway. Protein Cell.

[6] Shi X.B., Kachirskaia L., Yamaguchi H., West L.E., Wen H., Wang E.W., Dutta S., Appella E., Gozani O. (2007). Modulation of p53 function by SET8-mediated methylation at lysine 382. Mol. Cell.

[7] Jansson M., Durant S.T., Cho E.C., Sheahan S., Edelmann M., Kessler B., La Thangue N.B. (2008). Arginine methylation regulates the p53 response. Nat. Cell Biol..

[8] Bedford M.T., Clarke S.G. (2009). Protein Arginine Methylation in Mammals: Who, What, and Why. Mol. Cell.

[9] Guccione E., Richard S. (2019). The regulation, functions and clinical relevance of arginine methylation. Nat. Rev. Mol. Cell Biol..

[10] Jarrold J., Davies C.C. (2019). PRMTs and Arginine Methylation: Cancer’s Best-Kept Secret?. Trends Mol. Med..

[11] Yang Y., Bedford M.T. (2013). Protein arginine methyltransferases and cancer. Nat. Rev. Cancer.

[12] Tang J., Frankel A., Cook R.J., Kim S., Paik W.K., Williams K.R., Clarke S., Herschman H.R. (2000). PRMT1 is the predominant type I protein arginine methyltransferase in mammalian cells. J. Biol. Chem..

[13] Yamagata K., Daitoku H., Takahashi Y., Namiki K., Hisatake K., Kako K., Mukai H., Kasuya Y., Fukamizu A. (2008). Arginine Methylation of FOXO Transcription Factors Inhibits Their Phosphorylation by Akt. Mol. Cell.

[14] Zhao X., Jankovic V., Gural A., Huang G., Pardanani A., Menendez S., Zhang J., Dunne R., Xiao A., Erdjument-Bromage H. (2008). Methylation of RUNX1 by PRMT1 abrogates SIN3A binding and potentiates its transcriptional activity. Genes Dev..

[15] Liu L.M., Sun W.Z., Fan X.Z., Xu Y.L., Cheng M.B., Zhang Y. (2019). Methylation of C/EBPa by PRMT1 Inhibits Its Tumor-Suppressive Function in Breast Cancer. Cancer Res..

[16] Sachs N., de Ligt J., Kopper O., Gogola E., Bounova G., Weeber F., Balgobind A.V., Wind K., Gracanin A., Begthel H. (2018). A Living Biobank of Breast Cancer Organoids Captures Disease Heterogeneity. Cell.

[17] Yan L., Yan C., Qian K., Su H., Kofsky-Wofford S.A., Lee W.C., Zhao X., Ho M.C., Ivanov I., Zheng Y.G. (2014). Diamidine compounds for selective inhibition of protein arginine methyltransferase 1. J. Med. Chem..

[18] Zhang Y., Zhang Y.J., Zhao H.Y., Zhai Q.L., Zhang Y., Shen Y.F. (2014). The impact of R213 mutation on p53-mediated p21 activity. Biochimie.

[19] Cheng M.B., Zhang Y., Cao C.Y., Zhang W.L., Zhang Y., Shen Y.F. (2014). Specific phosphorylation of histone demethylase KDM3A determines target gene expression in response to heat shock. PLoS Biol..

[20] Liu G.F., Lu J.Y., Zhang Y.J., Zhang L.X., Lu G.D., Xie Z.J., Cheng M.B., Shen Y.F., Zhang Y. (2016). C/EBPalpha negatively regulates SIRT7 expression via recruiting HDAC3 to the upstream-promoter of hepatocellular carcinoma cells. Biochim. Biophys. Acta.

[21] Schafer M.J., White T.A., Iijima K., Haak A.J., Ligresti G., Atkinson E.J., Oberg A.L., Birch J., Salmonowicz H., Zhu Y. (2017). Cellular senescence mediates fibrotic pulmonary disease. Nat. Commun..

[22] Zhang B., Wu Q., Li B., Wang D., Wang L., Zhou Y.L. (2020). m(6)A regulator-mediated methylation modification patterns and tumor microenvironment infiltration characterization in gastric cancer. Mol. Cancer.

[23] Hu X., Wu L., Liu B., Chen K. (2021). Immune Infiltration Subtypes Characterization and Identification of Prognosis-Related lncRNAs in Adenocarcinoma of the Esophagogastric Junction. Front. Immunol..

[24] Wang H.B., Huang Z.Q., Xia L., Feng Q., Erdjument-Bromage H., Strahl B.D., Briggs S.D., Allis C.D., Wong J.M., Tempst P. (2001). Methylation of histone H4 at arginine 3 facilitating transcriptional activation by nuclear hormone receptor. Science.

[25] McBride A.E., Weiss V.H., Kim H.K., Hogle J.M., Silver P.A. (2000). Analysis of the yeast arginine methyltransferase Hmt1p/Rmt1p and its in vivo function—Cofactor binding and substrate interactions. J. Biol. Chem..

[26] Teyssier C., Ma H., Emter R., Kralli A., Stallcup M.R. (2005). Activation of nuclear receptor coactivator PGC-1alpha by arginine methylation. Genes Dev..

[27] Zhang X., Cheng X. (2003). Structure of the predominant protein arginine methyltransferase PRMT1 and analysis of its binding to substrate peptides. Structure.

[28] Cao L., Li W., Kim S., Brodie S.G., Deng C.X. (2003). Senescence, aging, and malignant transformation mediated by p53 in mice lacking the Brca1 full-length isoform. Genes Dev..

[29] Fujita K., Mondal A.M., Horikawa I., Nguyen G.H., Kumamoto K., Sohn J.J., Bowman E.D., Mathe E.A., Schetter A.J., Pine S.R. (2009). p53 isoforms Delta133p53 and p53beta are endogenous regulators of replicative cellular senescence. Nat. Cell Biol..

[30] Brady C.A., Jiang D., Mello S.S., Johnson T.M., Jarvis L.A., Kozak M.M., Kenzelmann Broz D., Basak S., Park E.J., McLaughlin M.E. (2011). Distinct p53 transcriptional programs dictate acute DNA-damage responses and tumor suppression. Cell.

[31] Zhou Y., Zhou B., Pache L., Chang M., Khodabakhshi A.H., Tanaseichuk O., Benner C., Chanda S.K. (2019). Metascape provides a biologist-oriented resource for the analysis of systems-level datasets. Nat. Commun..

[32] Xie C., Mao X., Huang J., Ding Y., Wu J., Dong S., Kong L., Gao G., Li C.Y., Wei L. (2011). KOBAS 2.0: A web server for annotation and identification of enriched pathways and diseases. Nucleic Acids Res..

[33] Pavlidis P., Noble W.S. (2003). Matrix2png: A utility for visualizing matrix data. Bioinformatics.

[34] An W., Kim J., Roeder R.G. (2004). Ordered cooperative functions of PRMT1, p300, and CARM1 in transcriptional activation by p53. Cell.

[35] Lee Y.J., Chang W.W., Chang C.P., Liu T.Y., Chuang C.Y., Qian K., Zheng Y.G., Li C. (2019). Downregulation of PRMT1 promotes the senescence and migration of a non-MYCN amplified neuroblastoma SK-N-SH cells. Sci. Rep..

[36] Jung S.Y., Li Y., Wang Y., Chen Y., Zhao Y., Qin J. (2008). Complications in the assignment of 14 and 28 Da mass shift detected by mass spectrometry as in vivo methylation from endogenous proteins. Anal. Chem..

[37] Gao J.J., Aksoy B.A., Dogrusoz U., Dresdner G., Gross B., Sumer S.O., Sun Y.C., Jacobsen A., Sinha R., Larsson E. (2013). Integrative Analysis of Complex Cancer Genomics and Clinical Profiles Using the cBioPortal. Sci. Signal..

[38] Cerami E., Gao J., Dogrusoz U., Gross B.E., Sumer S.O., Aksoy B.A., Jacobsen A., Byrne C.J., Heuer M.L., Larsson E. (2012). The cBio cancer genomics portal: An open platform for exploring multidimensional cancer genomics data. Cancer Discov..

[39] Razavi P., Chang M.T., Xu G., Bandlamudi C., Ross D.S., Vasan N., Cai Y., Bielski C.M., Donoghue M.T.A., Jonsson P. (2018). The Genomic Landscape of Endocrine-Resistant Advanced Breast Cancers. Cancer Cell.

