# Supplementary material for: Arginine Methyltransferase PRMT1 Regulates p53 Activity in Breast Cancer
Source: Life (Basel). 2021 Aug 5;11(8):789. doi: 10.3390/life11080789 (PMC8400051; doi:10.3390/life11080789)
Supplement: Supplementary file 1 [file life-11-00789-s001.zip › Supplementary/Supplementary Table S1.pdf]

Table S1. Primers used in this paper.

Primers for PRMT1(G80R)

	Sequence (5′-3′)
PRMT1(G80R)-F	CTGGACGTCGGCTCGCGCACCGGCATCCTCTGC
PRMT1(G80R)-R	GCAGAGGATGCCGGTGCGCGAGCCGACGTCCAG

RT-PCR Primers

	Sequence (5′-3′)
p21-F	GGCAGACCAGCATGACAGATT
p21-R	GCGGATTAGGGCTTCCTCTT
p53-F	CCCAAGCAATGGATGATTTGA
p53-R	GGCATTCTGGGAGCTTCATCT
GADD45A-F	CTGGAGGAAGTGCTCAGCAAAG
GADD45A-R	AGAGCCACATCTCTGTCGTCGT
PUMA-F	CGGAGACAAGAGGAGCAG
PUMA-R	GGAGTCCCATGATGAGATTG
BID-F	CCTTGCTCCGTGATGTCTTTC
BID-R	GTAGGTGCGTAGGTTCTGGT
PRMT1-F	TACACGCACTGGAAGCAGA
PRMT1-R	GGTTGTTCTTGGCGTTGG
GAPDH-F	GGTCGTATTGGGCGCCTGGTCACC
GAPDH-R	CACACCCATGACGAACATGGGGGC

shRNA sequences

	Sequence (5′-3′)
shPRMT1-1-F	CCGGCTTACCGCAACTCCATGTTTCCTCGAGGAAACATGGAGTTGCGGTAAGTTTTTG
shPRMT1-1-R	AATTCAAAAACTTACCGCAACTCCATGTTTCCTCGAGGAAACATGGAGTTGCGGTAAG
shPRMT1-2-F	CCGGTTGACTCCTACGCACACTTTGCTCGAGCAAAGTGTGCGTAGGAGTCAATTTTTG
shPRMT1-2-R	AATTCAAAAATTGACTCCTACGCACACTTTGCTCGAGCAAAGTGTGCGTAGGAGTCAA
