# Supplementary material for: Arginine Methyltransferase PRMT1 Regulates p53 Activity in Breast Cancer
Source: Life (Basel). 2021 Aug 5;11(8):789. doi: 10.3390/life11080789 (PMC8400051; doi:10.3390/life11080789)

Figure 1B top

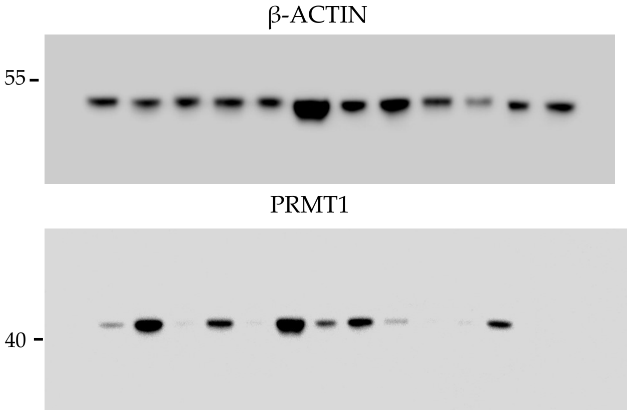


Figure 1B bottom

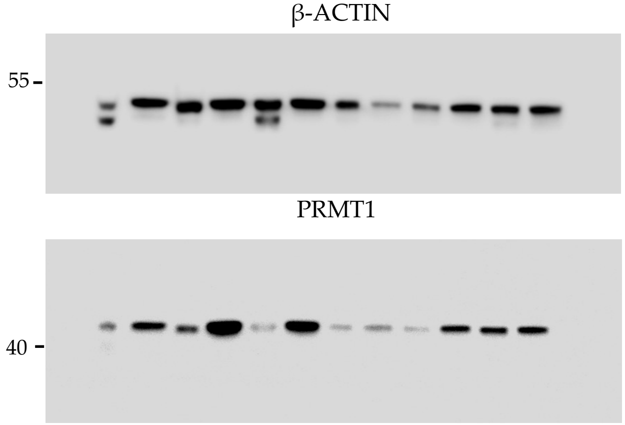


Figure 1D left

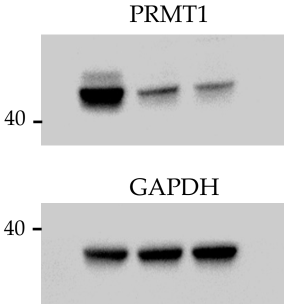


Figure 1D right

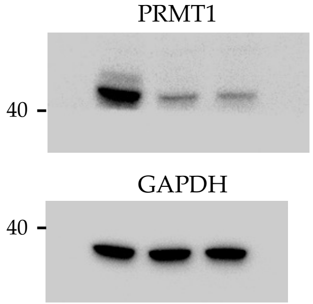


Figure 1H

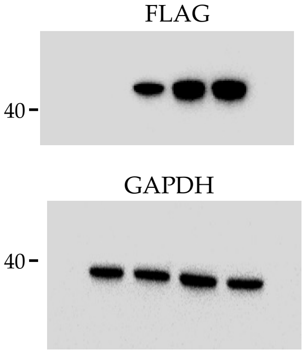


Figure 2E

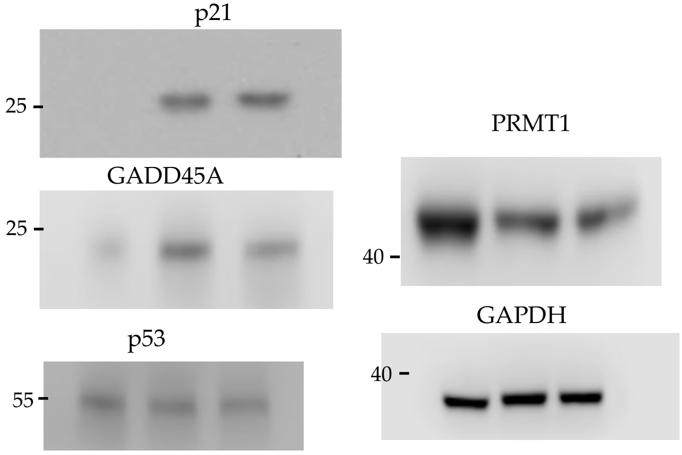


Figure 2F

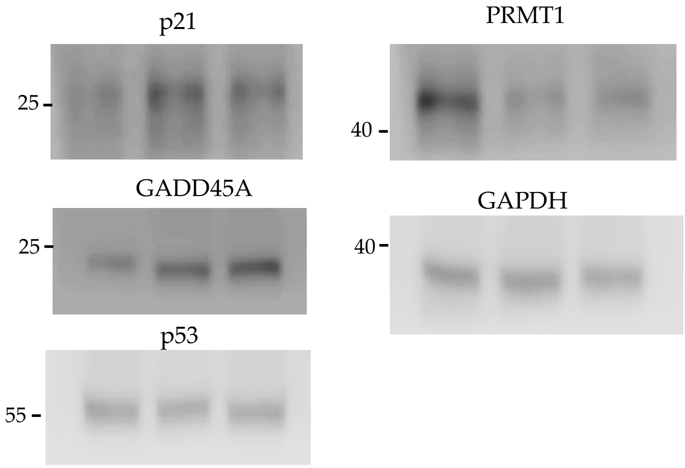

Figure 2G

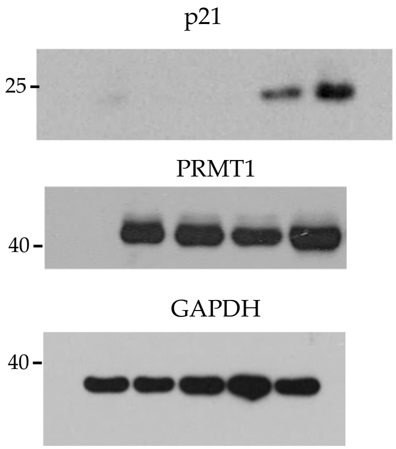


Figure 2H

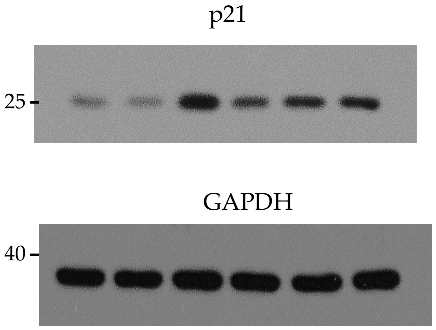


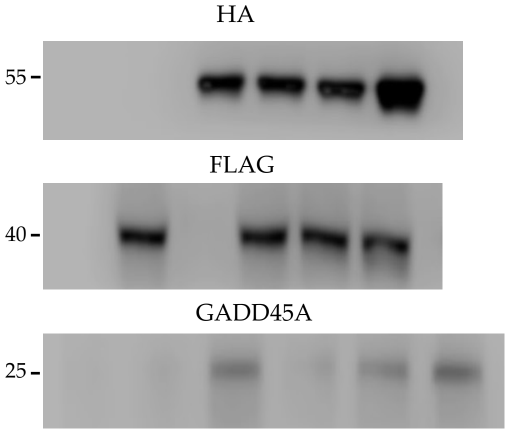


Figure 3A

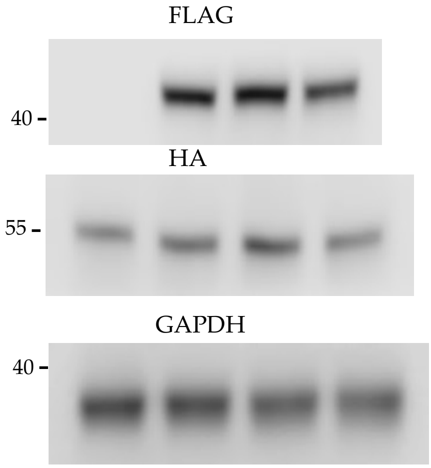


Figure 3B

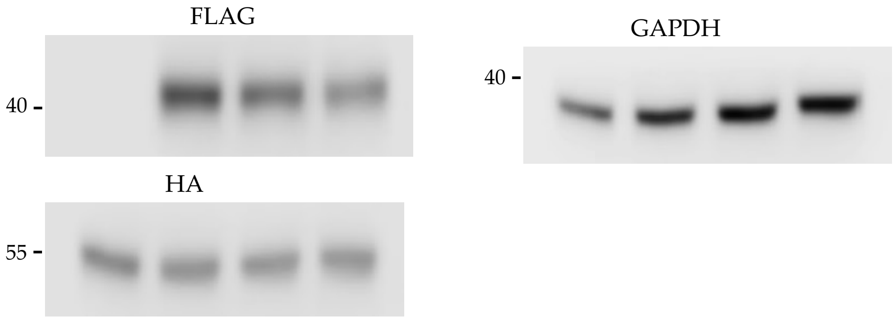


Figure 3C

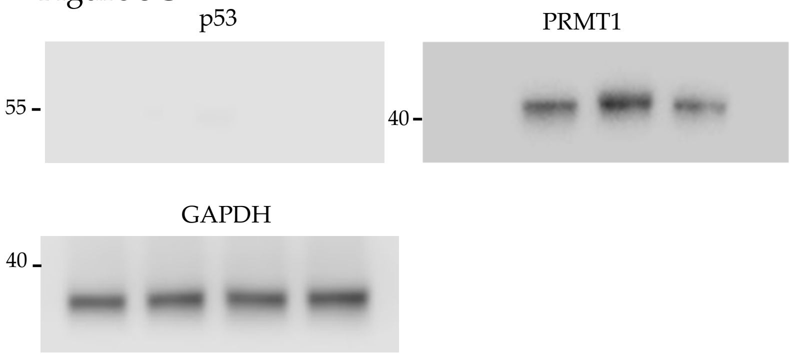


Figure 3D

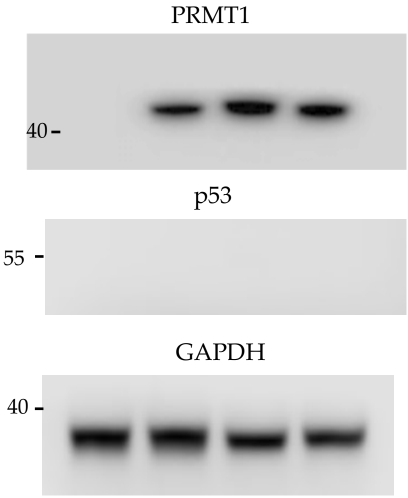

Figure 4A

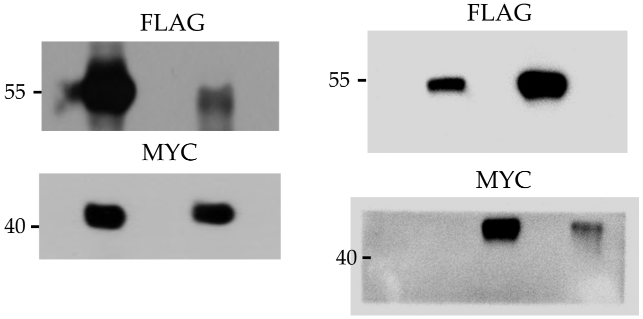


Figure 4B

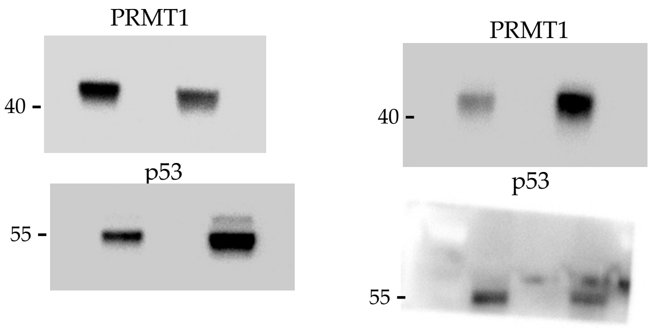


Figure 4C

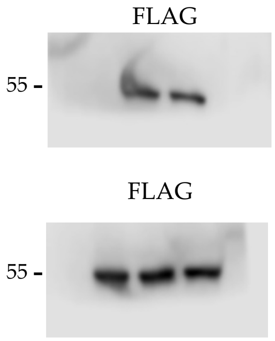


Figure 4D

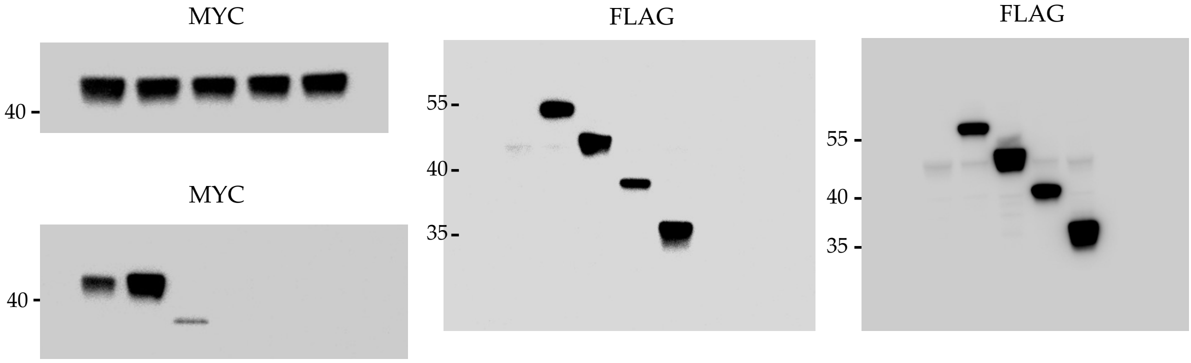

Figure 4E

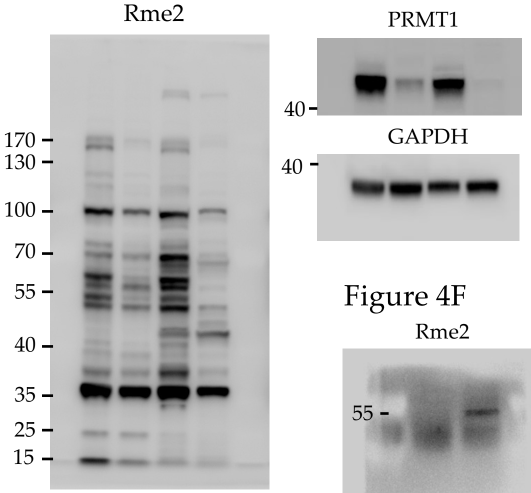


Figure 4F

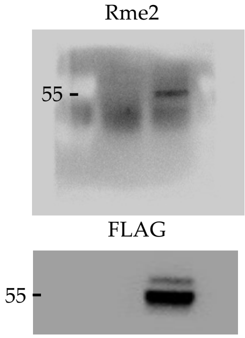


Figure 4G

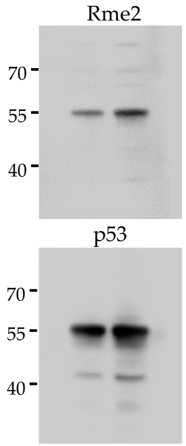


Figure 4H

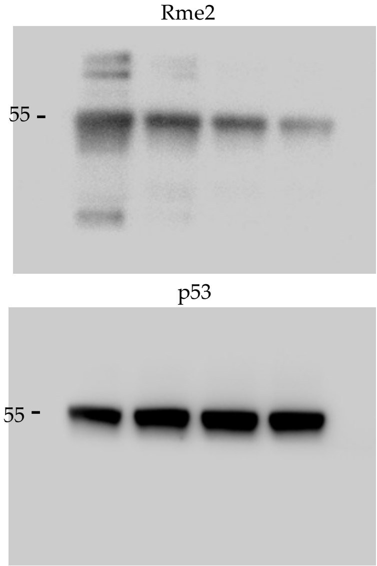


Figure 4J

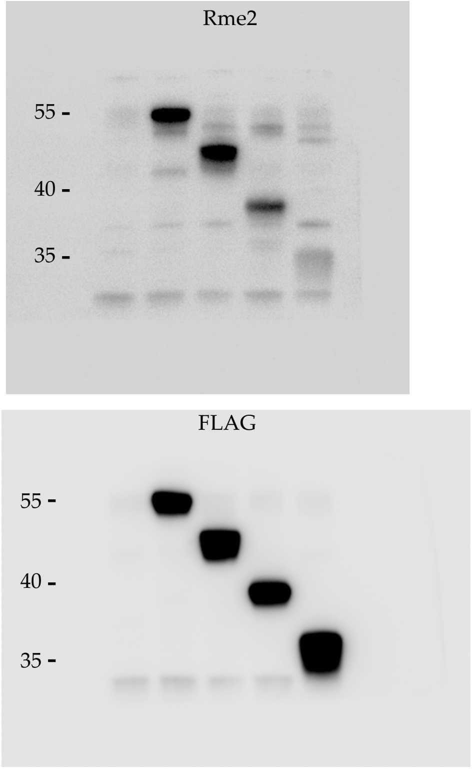


Figure 4K

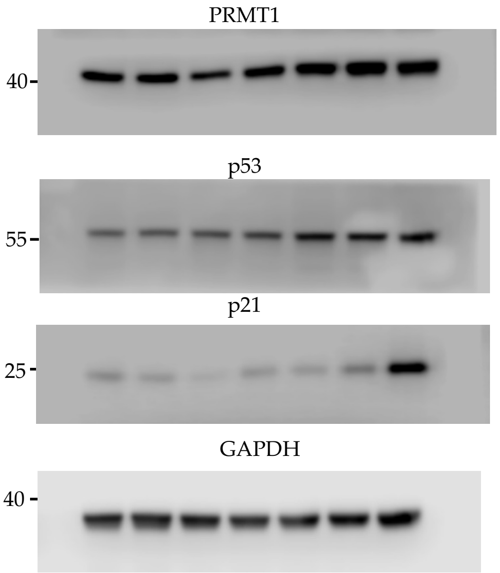


Figure 4I

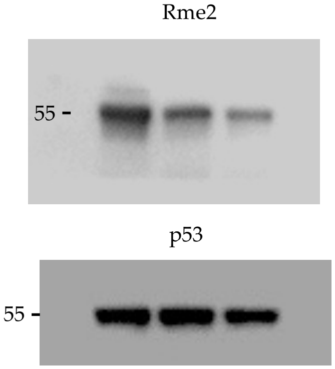


Figure 4L

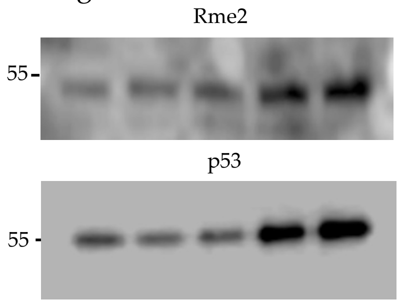

Figure 5A

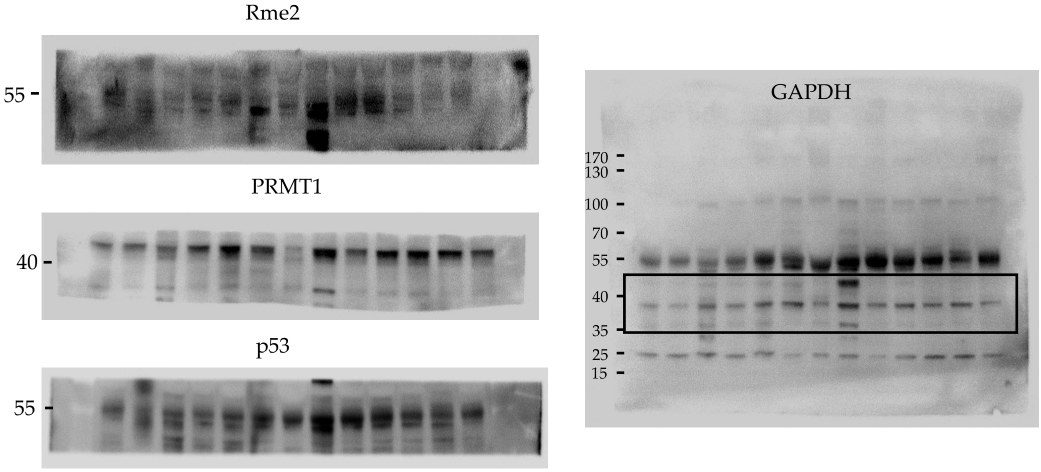


Figure 5B left

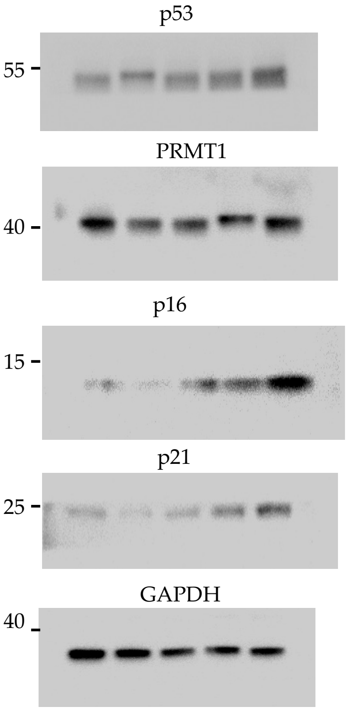


Figure 5B right

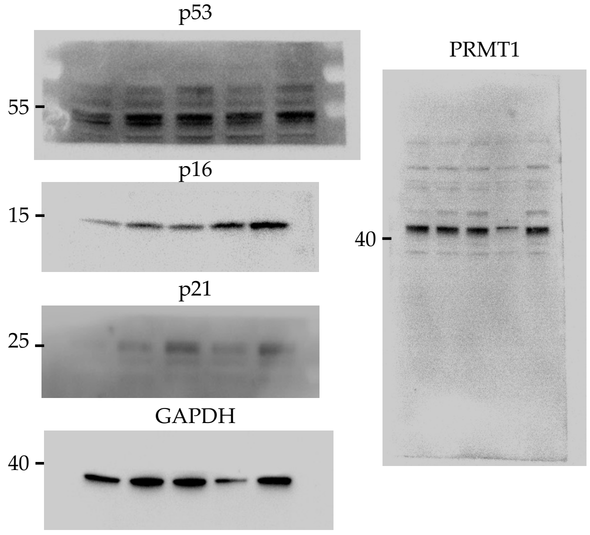


Figure 5C

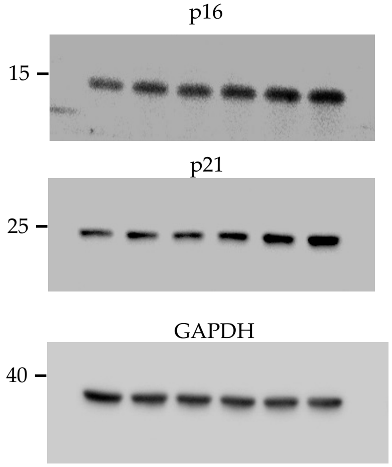


Figure 5D

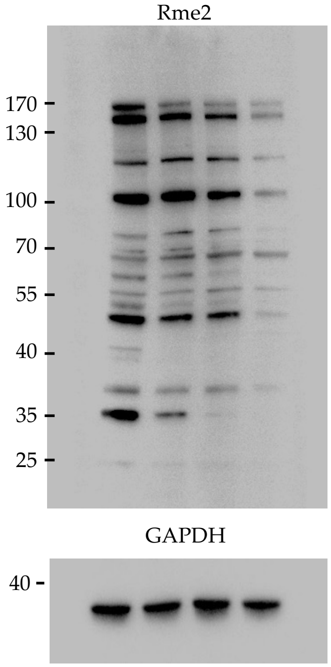

Supplement: Supplementary file 1 [file life-11-00789-s001.zip › Supplementary/Supplementary File S1.pdf]
