# Supplementary material for: Arginine Methyltransferase PRMT1 Regulates p53 Activity in Breast Cancer
Source: Life (Basel). 2021 Aug 5;11(8):789. doi: 10.3390/life11080789 (PMC8400051; doi:10.3390/life11080789)
Supplement: Supplementary file 1 [file life-11-00789-s001.zip › Supplementary/Supplementary Figure S1.pdf]

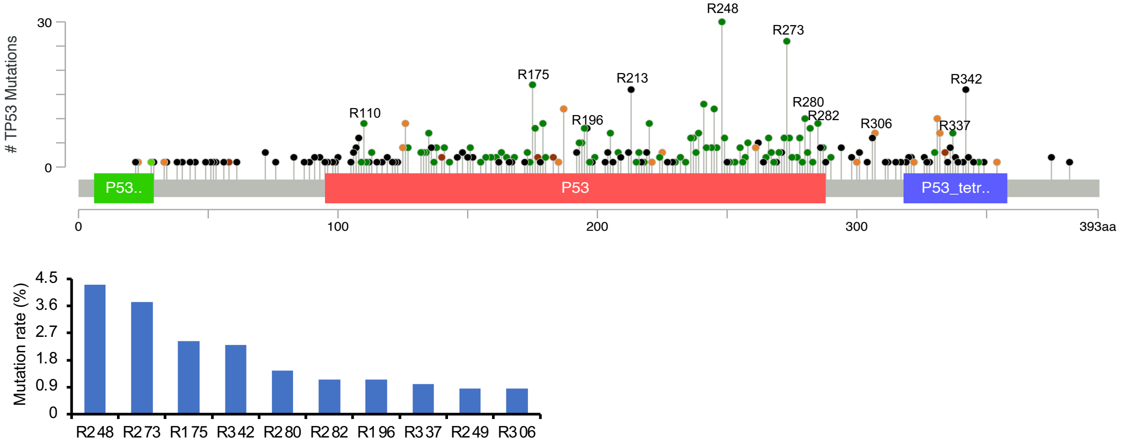

Figure S1. Mutation analysis of p53 gene in breast cancer patients. 1756 breast cancer patients/1989 samples were analyzed using cBioPortal. 696 mutations were found (top), and the mutation rates of arginine residues were shown (bottom).
